# A study of some psychological variables as predictors of COVID-19 vaccination anxiety among Faculty Members of Ain Shams University

**DOI:** 10.1038/s41598-024-75360-x

**Published:** 2024-11-04

**Authors:** Nashwa Abdel Monem Al Baseer, Hayam Saber Shaheen

**Affiliations:** Department of Psychology, Faculty of Women for Arts, Science and Education, Ain Shams University, 1 Asmaa Fahmy Street, Heliopolis, P.O. 11577, Cairo, Egypt

**Keywords:** Psychology, Risk factors

## Abstract

The study aimed to investigate the relative contribution of each of psychasthenia, psychological flexibility, suggestibility, and conspiracy beliefs in predicting the anxiety of vaccination with the anti COVID-19 vaccine among faculty members at Ain Shams University (ASU). Examining the difference in the sample’s scores on the vaccination anxiety scale and its sub-factors according to the variables (age—chronic diseases—academic specialization—previous infection with COVID-19—gender), the sample of the study included 139 faculty members at Ain Shams University aged ranged from 23 to 78 years, with a mean of 49.52 years, and a standard deviation of 13.29 years. The vaccination anxiety scale, psychasthenia scale, psychological flexibility scale, suggestibility scale, and conspiracy beliefs scale were used to collect data. The results revealed that the variables of suggestibility and conspiracy beliefs significantly contribute to predicting the vaccination anxiety among faculty members at Ain Shams University. There is a significant difference in the average scores on the vaccination anxiety scale according to age, with the difference in favor of the age group less than fifty years, as well as according to the presence/absence of a previous infection with COVID-19, with the vaccination anxiety being higher for those who were previously infected. There has been no observed significant difference according to the presence/absence of chronic diseases or academic specialization.

The first COVID-19 cases were recorded in China in the city of Wuhan. The infections spread throughout the following months around the world, and by March 2020, about 9,125,495 cases were reported to the World Health Organization as the spread of the pandemic expanded, including 232,433 deaths, and this was accompanied by the beginning of recording neuropsychiatric complications^1,2^.

The pandemic also sparked widespread anxiety, particularly concerning the COVID-19 vaccine, fueled by rumors that dampened global vaccination efforts. In psychological literature, COVID-19 vaccination anxiety mirrors health anxiety, involving fear of illness and excessive concern about vaccine safety and efficacy^3^. Many studies indicate a clear global concern about COVID-19 vaccines underscores the need for extensive data and information^4,5^.

One of the most prominent problems associated with COVID-19 is the anxiety related to its anti-vaccine, the extent of its effectiveness, and whether it has future side effects. In rare cases, the damage can be serious, a fact that has caused many people to have more anxiety and reluctance to take the vaccine, especially since every period a new mutant appears, which increases anxiety about the effectiveness of the vaccines in circulation^6^.

Also, a lack of confidence in the effectiveness of vaccination, a conspiracy belief that negatively affected the demand for vaccination especially with the presence of procedures that imposed compulsory vaccination. The speed of developing vaccines is one of the factors that contributed to this concern and the possibility that this is linked to a number of psychological variables^7^.

Gerretsen et al., showed that anxiety and hesitation about vaccination stemmed from concerns about: 1) confidence in the vaccine, 2) satisfaction with the vaccine, 3) demographic variables and other psychological factors^8^.

It was found that resistance to the COVID-19 vaccine in the United Kingdom and the Republic of Ireland was associated with a lack of trust in experts and authority figures (i.e. scientists, healthcare professions, government), religious and conspiratorial beliefs, anti-immigrant views, and lower levels of agreeableness, conscientiousness, and emotional stability^9^.

Personality traits are among the important psychological vulnerability factors that influence the way any given individual reacts to a viral outbreak, including COVID-19^10^. Personality traits might affect how individuals handle COVID-19-related anxiety. For instance, neuroticism is a trait that may capture people’s tendency to avoid risk^11^.

Cultural and social heritage might affect how individuals’ psychological reactions and behaviors in response to COVID-19 vaccination anxiety. It is impacted with their personal, cultural and social reasons, like personal perceptions related to the likelihood of exposure to disease-causing agents, the severity of the disease, the safety and efficacy of the vaccine, and the novelty of the vaccine’s development^9^.

The current research can enhance understanding the reasons for vaccine anexity among university faculty members that represent a highly educated and culturally influential segment of society, serving as role models within the community regarding the COVID-19 vaccine. Shedding light on various psychological, demographic, cultural and social factors associated with vaccination anxiety, which can play a crucial role in their academic and research activities.

Therefore, the current research intends to study several significant psychological variables identified as psychasthenia, psychological flexibility, suggestibility, and conspiracy beliefs. Additionally, demographic variables such as gender, academic major, chronic diseases, and previous COVID-19 infection that are supposed to play a role in and be associated with vaccination anxiety.

## Aim of the study

The aim was to investigate the relative contribution of psychasthenia, psychological flexibility, suggestibility, conspiracy beliefs in predicting COVID -19 vaccine’s vaccination anxiety among ASU faculty members; as well as to investigate the difference of sample scores on the vaccination anxiety scale and its sub – factors depending on various variables (age—chronic disease—academic major – previous COVID-19 infection—Gender).

### The importance of this research stems from several factors

A. The significance of this research stems from its sample; as it represents university faculty members, an important segment and a broad sector of society that needs more attention and care, which reflects positively not only on the individual level but also on the general community.
B. This research derives its importance from its variables; as the variables of psychasthenia, psychological flexibility, suggestibility, and conspiracy beliefs are of great importance, especially in their relation to COVID -19 vaccination anxiety among university faculty members. Thier work often involves interacting with increasing numbers of students, which facilitates the transmission of infection.
C. This research may have economic significance; as addressing the anxiety of university faculty members regarding COVID -19 vaccine uptake could improve the services provided to them and maximize the benefits of vaccination, which may contribute to saving a lot of the financial costs that may be incurred by refusing vaccination and the subsequent spread of the pandemic.
D. There is no Arabic study to the extent of the researchers’ knowledge that has focused on the variables of the current study collectively among Staff University in an attempt to understand the nature of the relationship between them, and therefore to provide recommendations that benefit healthcare systems and those involved in completing the process of COVID-19 vaccination.
E. This research involves preparing four scales to assess COVID-19 vaccine anxiety, psychasthenia, suggestibility, and conspiracy beliefs; which may contribute to enriching the library of psychological measurement tools.

## Methodology and procedures

Participants were invited through a link via Google Form from 6 March to 7 August (2021). Through this portal, faculty members and teaching assistants from the Ain Shams University community could voluntarily participate in the study based on their interests after reviewing the study’s title, description, and objective. No rewards were offered for participation. Participants with internet access could participate in the study. The inclusion criteria for all participants were: (1) being a teaching assistant to a professor; and (2) having no missing values in the online survey. The survey was hosted on the Google Form platform for nearly five months, and the link was distributed through the university’s official channels via WhatsApp, the most commonly used social media platform in the workplace in Egypt. Upon receiving and clicking the electronic link, participants were directed to information about the study and consent. At the beginning of the survey, participants were informed that their data would be anonymous and confidential and that participation was voluntary. After agreeing to participate in the survey, participants provided socio-demographic details (such as age, gender, academic specialization, previous COVID-19 infection, and chronic illnesses). Subsequently, a set of questions appeared in five sections sequentially.

The research method: the research was based on the comparative correlational descriptive method to detect the contribution of psychasthenia, psychological flexibility, suggestibility, and conspiracy beliefs in predicting.

COVID-19 vaccine anxiety among faculty members at ASU. Examining the difference of sample scores on the vaccination anxiety scale and its sub – factors depending on different variables (age—chronic disease – academic major – previous COVID -19 infections – gender). Our research confirms that all research procedures were performed in accordance with relevant guidelines/regulations from the Ethics Committee at Ain Shams University.

### Sampling

Study sample was selected using accidental sampling and included the following:

A. The sample used to assess the psychometric competence of the study tools consisted of 100 faculty members at ASU. The participants’ ages ranged from 23 to 78 years, with an average of 48.64 years and a standard deviation of 13.62.
B. The sample of the descriptive study included 139 faculty members from various faculties of ASU, their ages ranged from 23-78 years with an average (49.52) and a standard deviation (13.29) years, and their specifications were as shown in Table 1.

**Table 1:** Demographic characteristics of participants (N = 139).

Variable	*n* (%)
Gender	
Male	20 (14.4)
Female	119 (85.6)
Academic major	
Literary	92 (66.2)
Scientific	47 (33.8)
Chronic diseases	
With	58 (41.7)
Non	81 (58.3)
Previous COVID-19 Infection	
Yes	59 (42.4)
No	80 (57.6)

### Study tools

The study scales were prepared according to the following steps:

**First:** A review of the theoretical frameworks and previous studies related to the study variables was conducted with the aim of defining them operationally and identifying their aspects. Additionally, the available scales were reviewed, **Second:** Determining the components of the scales, **Third:** Formulating the scales items: Based on the results of the previous step.

***The COVID-19 vaccination anxiety scale*** (prepared by the researchers): It contains (30) items divided into three factors: anxiety related to confidence in the effectiveness of the vaccine (13 items), anxiety related to professional, social, and family responsibility (6 items), emotional symptoms of anxiety (11 items), that are scored three point Likert scale ranging from 3 = always agree, 2 = sometimes agree, 1 = rarely agree, in the case of positive items, and are reverse-scored in the case of negative items. Total scores on this scale range from 30 to 90. High scores indicate high levels of vaccination anxiety.

Psychometric efficiency of the scale, reliability; which was tested by the split-half (0.83), Cronbach’s α 0.94 for the whole scale, Cronbach’s α for factors were 0.90 about the anxiety associated with confidence in the effectiveness of the vaccine, and 0.73 about the anxiety associated with professional, social and family responsibility, and 0.88 for the emotional symptoms of anxiety. As for validity; it was verified by the validity of the expert judges who are instructed to examine if the construct has been adequately covered by the items generated, and the Factorial analysis validity.

Table 2 presents the exploratory factor analysis was conducted using Hotelling’s principal component analysis with Varimax rotation and employing Kaiser’s criterion (eigenvalues greater than one). The Varimax rotation resulted in three factors, as shown in the component matrix after rotation. The items loaded purely on the three factors of the scale, with positive loadings. A loading of 0.3 or higher was considered adequate according to Guilford’s criterion.

**Table 2:** Exploratory factor analysis of the COVID-19 vaccine anxiety scale.

First component		Second component		Third component	
Item	Loading	Item	Loading	Item	Loading
Vaccination with the COVID-19 vaccine does not prevent infection with the virus	0.551	I worry about my professional future due to vaccination	0.733	I fear that I might get infected with the virus despite vaccination	0.304
I doubt the government’s ability to combat the coronavirus	0.766	Dealing with large crowds in lectures is terrifying even after vaccination	0.739	Signing a form where I take responsibility for any consequences of vaccination is terrifying in itself	0.449
I am concerned about the potential negative consequences of the vaccination	0.660	I tremble at the mere thought of vaccinating my children	0.719	I do not feel safe about getting vaccinated	0.610
I lose confidence in the vaccine’s effectiveness due to its variety and the different samples tested	0.824	I feel a great social responsibility toward my family, which makes me enthusiastic about vaccination	0.448	I know how to control my fears toward mandatory vaccination	0.620
The new virus variants make me lose faith in the usefulness of any vaccine	0.739	I fear losing a family member due to the consequences of the vaccine	0.446	I am worried about the side effects of the vaccine	0.678
I lose confidence in the healthcare system’s ability to handle the possible consequences of the vaccination	0.817	I fear for my health because of the consequences of the vaccine	0.529	I feel irritable since the beginning of the discussion about mandatory vaccination against COVID-19	0.728
Humanity is headed toward disasters due to the spread of epidemics	0.434			I find it difficult to control my anxiety about the coronavirus and its consequences	0.553
I doubt the vaccine’s effectiveness due to the lack of clear data about it	0.819			I get stressed when I hear about someone dying after getting vaccinated	0.799
I lose confidence in the vaccine’s effects due to conflicting information about its impact	0.740			I am overwhelmed by feelings of fear due to excessive thinking about illness and death after vaccination	0.694
I fear attending social gatherings despite being vaccinated	0.719			I get anxious just thinking about the vaccination	0.777
I doubt the vaccine with any change in my body	0.580			I am disturbed by the numerous rumors regarding the vaccine’s effectiveness	0.678
The idea of vaccination causes me stress	0.396				
I expect future health problems after getting vaccinated	0.620				

As shown in Table 2, it is evident from the factor analysis after rotation that the first factor had an eigenvalue of 6.87, explaining 22.92% of the variance in the scale scores, with 13 items loading onto it. This factor can be named "anxiety related with vaccine confidence" The second factor had an eigenvalue of 6.71, explaining 22.38% of the variance in the scale scores, with 6 items loading onto it. This factor can be named " Anxiety related to professional, social, and family responsibilities " The third factor had an eigenvalue of 2.66, explaining 8.89% of the variance in the scale scores, with 11 items loading onto it. This factor can be named Emotional symptoms of anxiety". Thus, the total explained variance by these factors is 54.20%, which is a reasonable value of explained variance. None of the scale items were excluded.

***The psychasthenia scale*** (prepared by the researchers): This scale contains (17 items) distributed over two factors: mental asthenia (7 items), and behavioral asthenia (10 items), that are scored three point Likert scale ranging from 3 = always agree, 2 = sometimes agree, 1 = rarely agree. All items were positively worded about psychasthenia. Total scores on this scale range from 17–51. High scores indicate high levels of psychasthenia. The reliability of the scale was verified by calculating the split-half, it (0.76), Cronbach’s *α* (0.92 for the whole scale, Cronbach’s *α* for both factors were 0.88 for mental asthenia, and 0.89 for behavioral asthenia). As for validity; it was verified by the validity of the expert judges who are instructed to examine if the construct has been adequately covered by the items generated, and the factorial analysis validity.

Table 3 presents the exploratory factor analysis conducted using Hotelling’s principal component analysis with Varimax rotation and employing Kaiser’s criterion (eigenvalues greater than one).

**Table 3:** Exploratory factor analysis of the COVID-19 psychasthenia scale.

First component		Second component	
Item	Loading	Item	Loading
A set of thoughts related to the vaccine dominates my mind, even though I know they are trivial.	0.735	I avoid using public restrooms for fear of infection.	0.391
The idea of death and illness takes over my mind with the spread and mutation of the pandemic.	0.745	I constantly check the sanitation measures at the office and home.	0.576
I focus on the smallest details about the vaccine to the point of exhaustion.	0.759	I wash soap multiple times before using it, fearing contamination.	0.687
I am a compulsive person.	0.582	I wash my hands several times, even without touching anything.	0.729
My mind is constantly preoccupied with the effects of the vaccine and virus mutations.	0.756	I consume a large amount of disinfectants.	0.776
I obsessively and involuntarily dwell on certain thoughts about the vaccine.	0.789	I wash my hands after shaking hands with anyone.	0.765
The idea of the end of the world takes over after seeing the infection and death rates of COVID-19.	0.681	Every time I go out, I wash my clothes immediately upon returning home.	0.773
		I avoid shaking hands with anyone for fear of infection.	0.763
		I wash vegetables with water and soap multiple times.	0.736
		Caution plays a significant role in surviving the virus.	0.512

As shown in Table 3, it is evident from the factor analysis after rotation that the first factor had an eigenvalue of 4.92, accounting for 28.96% of the variance in the scale scores. This factor was loaded with 7 items and can be labeled as “mental asthenia”. The second factor had an eigenvalue of 4.40, explaining 25.91% of the variance, and was loaded with 10 items. This factor can be termed “behavioral asthenia”. The total variance explained by these factors was 54.87%, which is a reasonable value of explained variance. None of the scale items were excluded.

***Psychological flexibility scale*** (Connor and Davidson^12^): This scale contains (25 items), that are scored three point Likert scale ranging from 3 = always agree, 2 = sometimes agree, 1 = rarely agree, in the case of positive items, and are reverse-scored in the case of negative items. Total scores on this scale range from 25 to 75. High scores indicate high levels of psychological flexibility. The reliability of the scale verified by calculating the split-half, it reached (0.88), and the Cronbach’s *α* (0.94).

Table 4 presents the exploratory factor analysis conducted using Hotelling’s principal component analysis with Varimax rotation and employing Kaiser’s criterion (eigenvalues greater than one).

**Table 4:** Exploratory factor analysis of the COVID-19 psychological flexibility scale.

First component		Second component		Third component	
Item	Loading	Item	Loading	Item	Loading
Able to adapt to change	0.417	Best effort no matter what	0.474	Can handle unpleasant feelings	0.675
Close and secure relationships	0.345	You can achieve your goals	0.706	Have to act on a hunch	0.587
Sometimes fate or God can help	0.335	When things look hopeless, I don’t give up	0.624	Strong sense of purpose	0.782
Can deal with whatever comes	0.593	Know where to turn for help	0.553	In control of your life	0.818
Past success gives confidence for new challenge	0.677	Under pressure, focus and think clearly	0.628	I like challenges	0.809
See the humorous side of things	0.549	Prefer to take the lead in problem solving	0.708	You work to attain your goals	0.765
Coping with stress strengthens	0.499	Not easily discouraged by failure	0.746	Pride in your achievements	0.720
Tend to bounce back after illness or hardship	0.543	Think of self as strong person	0.805		
Things happen for a reason	0.445	Make unpopular or difficult decisions	0.711		

As shown in Table 4, it is evident from the factor analysis after rotation that, the eigenvalue is 10.12, explaining 40.48% of the variance in the scale scores. This is a reasonable value of explained variance. None of the scale items were excluded.

***The suggestibility scale*** (prepared by the researchers): This scale contains (18 items) distributed over three factors: persuasion (6 items), psychological reaction (6 items), and compliance (6 items), that are scored three point Likert-type scale ranging from 3 = always agree, 2 = sometimes agree, 1 = rarely agree. All items were positively worded about suggestability. Total scores on this scale ranged from 18 to 54. High scores indicate high levels of suggestability. The reliability of the scale was verified by the split-half, it was (0.85). The Cronbach’s *α* was (0.92) for the scale as a whole, it was (0.77; for the factors of the persuasion, 0.87, for the factor of the psychological reaction, and 0.85, for coping). As for validity, it was verified by the validity of the expert judges who are instructed to examine if the construct has been adequately covered by the items generated, and the Factorial analysis validity.

Table 5 presents the exploratory factor analysis conducted using Hotelling’s principal component analysis with Varimax rotation and employing Kaiser’s criterion (eigenvalues greater than one).

**Table 5:** Exploratory factor analysis of the suggestability scale.

First component		Second component		Third component	
Item	Loading	Item	Loading	Item	Loading
Traditional remedies are a substitute for the vaccine	0.677	My heart races when I think about the risks of vaccination	0.372	I repeat what others say about the futility of the government’s procedures against the COVID-19 pandemic	0.644
I follow others’ advice regarding the dangers of getting vaccinated	0.746	I feel fear when I see someone afraid of the effects of the vaccine	0.355	When I discuss vaccination, I repeat what I hear from those around me	0.700
I trust the information I get from those around me about the effectiveness of the vaccine	0.824	I get anxious about the increasing number of deaths despite vaccination	0.747	I hesitated to decide on vaccination after hearing the opinions of those around me	0.820
When someone talks to me about the risks of vaccination, I am influenced by their opinion	0.639	It is better to gain immunity naturally rather than through vaccination	0.821	I am influenced by the opinion that vaccination is just a hoax	0.561
I am influenced by what is reported in the media about the effectiveness of the vaccine	0.768	I get anxious when I hear others talk about the COVID-19 vaccine	0.785	It took me a long time to be convinced of the need for vaccination	0.638
The advice of others helped me decide to get vaccinated	0.576	I am influenced by those who claim that we might one day face a shortage of essential food supplies	0.363	I got vaccinated simply because my family members agreed to it	0.528

As shown in Table 5, it is evident from the factor analysis after rotation that, the eigenvalue of 4.50 and explains 25.00% of the variance in the scale scores. This factor is saturated with six items and can be labeled “Persuasion”. The second factor has an eigenvalue of 3.76 and explains 20.90% of the variance in the scale scores, and it can be labeled “Psychological Reaction”, with six items loading on it. The third factor has an eigenvalue of 2.62 and explains 14.58% of the variance in the scale scores, and it can be labeled “Compliance”, also with six items loading on it. The total variance explained by these factors is 60.48% which is a reasonable value of explained variance. None of the scale items were excluded.

***The conspiracy beliefs scale*** (prepared by the researchers): This scale contains (22 items) distributed over two factors: the first factor is the conspiracy beliefs related with the safety of the COVID – 19 vaccine (12 items), the second is the conspiracy beliefs related to the genesis of COVID-19 (10 items), and that are scored three point Likert scale ranging from 3 = always agree, 2 = sometimes agree, 1 = rarely agree, in the case of positive items, and were reversed in the case of negative items (No.6,10). Total scores on this scale ranged from 12 to 36. High scores indicate high levels of conspiracy beliefs. The reliability of the scale was verified by calculating the Split-half, it was (0.80), and Cronbach’s *α* was (0.89) (for the whole scale, and 0.82, for the conspiracy beliefs associated with the genesis of COVID-19, and 0.84, for the conspiracy beliefs associated with the safety of the COVID-19 vaccine). As for validity, it was verified by the validity of the expert judges who are instructed to examine if the construct has been adequately covered by the items generated, and the Factorial analysis validity.

Table 6 presents the exploratory factor analysis conducted using Hotelling’s principal component analysis with Varimax rotation and employing Kaiser’s criterion (eigenvalues greater than one).

**Table 6:** Exploratory factor analysis of conspiracy beliefs scale.

First component		Second component	
Item	Loading	Item	Loading
It was developed with the aim of causing sexual dysfunction to reduce the global population	0.572	COVID-19 an inevitable result of economic conflicts between major countries	0.764
Its safety data is fabricated	0.734	COVID-19 was manufactured to change the demographic composition of countries around the world	0.767
Its role in immunizing individuals is limited	0.818	It was Created to eliminate the elderly	0.358
Pharmaceutical companies are hiding its risks	0.693	Governments deliberately hide the truth about it from the general public	0.486
People are deceived about its effectiveness	0.683	It was spread in this way for certain purposes that will emerge later	0.798
People are deceived about its safety	0.806	It was a result of an unintended human error	0.745
It contains nanochips to control humans	0.722	A conspiracy orchestrated by major countries to dominate the world	0.742
The government is trying to cover up its link to many diseases	0.780	A conspiracy devised by global entities to make enormous profits from vaccines and other products	0.856
It has safety issues that could harm human health	0.805	It happened due to deliberate environmental pollution	0.546
It is designed to harm developing countries	0.745	It is not as severe as they promote it to be	0.683
All the news about its effectiveness is false	0.795		
It is an important tool to reduce the spread of infectious diseases	0.671		

As shown in Table 6, it is evident from the factor analysis after rotation that, the eigenvalue of the first factor has an eigenvalue of 7.24 and explains 32.93% of the variance in the scale scores, with 12 items loading on it. This factor can be named "Conspiracy Beliefs Related to Vaccine Safety". The second factor has an eigenvalue of 4.86 and explains 22.11% of the variance in the scale scores, with 10 items loading on it. This factor can be named "Conspiracy beliefs related to the genesis of COVID-19". Together, the two factors explain a total variance of 55.04% which is a reasonable value of explained variance. None of the scale items were excluded.

In preparing the scales for the study variables (vaccination anxiety, psychasthenia, suggestibility, and conspiracy beliefs), consideration was given to ensuring that they were linguistically and content-wise appropriate for the local culture of Egyptian society, including its religious beliefs and social norms that could influence acceptance or rejection of vaccination. This was verified by the validity of the expert judges.

### Statistical analysis

Statistical analyses were conducted using IBM Statistical Package for the Social Sciences (SPSS) for Windows. In light of the statistical description of the study variables, hypotheses, and sample size, parametric statistics were used, represented by the correlation coefficient, *t*-test for independent samples, the effect size, and multiple regression analysis^13^, and non parametric test (Mann–Whitney U test).

## Results

The Table 7 presents the descriptive statistics of the sample’s responses to the study scales**.**

**Table 7:** Descriptive statistics of the sample’s responses to the study scales.

	COVID-19 vaccine anxiety	Psychasthenia	Psychological flexibility	Suggestibility	Conspiracy beliefs
Minimum	30	10	39	18	26
Maximum	86	30	75	54	63
Mean	56.75	18.42	64.21	27.75	42.27
Medium	58	18	66	26	42
Std Deviation	13.63	5.12	8.81	7.53	8.85
Skewness	0.035	0.493	˗ 0.609	0.950	0.326
Std. Error of Skewness	0.206	0.206	0.206	0.206	0.206
Kurtosis	˗ 0.837	˗ 0.516	˗ 0.459	0.851	˗ 0.507
Std. Error of Kurtosis	0.408	0.408	0.408	0.408	0.408

**The first assumption**: "The anxiety of vaccination with the COVID-19 vaccine among faculty members at ASU can be predicted from their scores in psychological flexibility, psychasthenia, suggestibility, and conspiracy beliefs". To verify this assumption, a multiple regression analysis was conducted, and the results are shown in Table 8.

**Table 8:** Multiple regression analysis results showing the contribution values (*β*), "*F*" values, and statistical significance of variables (anxiety of vaccination with the anti-COVID-19 vaccine, psychasthenia, psychological flexibility, suggestibility and conspiracy beliefs) in predicting COVID-19 vaccine anxiety among faculty members at ASU.

	Sum of squares	Df	Mean square	F	Signifi-cance	Dependent variable	Model	Regression coefficient (B)	St. Error	Beta	t	Significance	Partial eta squared
Regression	12191.47	4	868.3047	30.359	0.001	COVID-19 Vaccine Anxiety	Psychasthnia	0.209	0.140	0.115	1.49	0.138	0.12
Residuals	13452.717	134	100.393				psychological flexibility	-0.167	0.099	-0.108	-1.69	0.09	− 0.14
Total	25644.187	138					Suggestibility	0.375	0.153	0.207	2.44	0.016	0.20
							conspiracy beliefs	0.793	0.110	0.515	7.18	0.001	0.52
Multiple Correlation Coefficient	0.689	Squared Multiple Correlation Coefficient (R²)	0.475	Adjusted Squared Multiple Correlation Coefficient	0.460		Constant		17.6	Standard Error of constant		7.55	

It is clear from the values recorded in Table 8 that the independent variables explain (47%) of the amount of variation in the dependent variable in statistical significance of (0.001). It also turns out that the values of the regression coefficient are not statistically significant for both the variables of psychasthenia and psychological flexibility; however, they are only statistically significant for the variables of suggestibility and conspiracy beliefs, at the level of significance (0.01, and 0.001). This indicates that these two variables statistically and significantly contribute to predicting COVID-19 vaccination anxiety among faculty members at ASU.

**The second assumption**: "the average scores of the study sample from faculty members at ASU on the COVID-19 vaccination anxiety scale and its sub-factors do not differ by age (less than 50 years—fifty years and older)". To verify this assumption, the (*t*) test for independent samples was used, and its results are listed in Table 9.

**Table 9:** Means, standard deviations, *t*-test values, and significance between faculty members on the scale of COVID vaccine anxiety and its sub-factors according to age.

Statistical values	< 50 years old (*n* = 72)		> 50 years old (*n* = 67)		t Value	Significance	D	Effect size
Variable	Mean	Standard deviation	Mean	Standard deviation				
Anxiety related with vaccine confidence	25.83	6.31	23.68	6.40	1.99	0.024	0.03	Small
Anxiety related to professional, social, and family responsibilities	11.68	2.86	10.16	2.91	3.09	0. 001	0.50	Medium
Emotional symptoms of anxiety	22.36	5.37	19.54	5.52	3.51	0.001	0.50	Medium
Total score	59.87	12.24	53.39	13.33	2.88	0. 002	0.50	Medium

It is clear from the values recorded in Table 9, it can be seen that there are statistically significant differences between the averages of the study sample scores on the COVID-19 vaccination anxiety scale and its sub-factors according to age, where the values of (t) ranged between (1.99 and 3.09), all of which are statistically significant, and the significance level ranged between (0.05) and (0.001).

**The third assumption**: "The average scores of the study sample from faculty members at ASU on the COVID-19 vaccination anxiety scale and its sub-factors do not differ depending on the affliction with chronic diseases or not". To verify this assumption, a ***t***-test was used for independent samples, and its results are listed in Table 10.

**Table 10:** Means, standard deviations, *t*-test values, and significance between faculty members afflicted with chronic diseases and non-afflicted on the scale of COVID-19 vaccine anxiety and its sub-factors.

Statistical values	Afflicted with chronic diseases (*n* = 81)		Non-afflicted (*n* = 58)		T Value	Significance	D	Effect size
Variable	Mean	Standard deviation	Mean	Standard deviation				
Anxiety related with Vaccine Confidence	24.34	6.76	25.12	6.189	0.704	0.241	0.01	Small
Anxiety related to professional, Social, and family responsibilities	10.34	2.95	11.38	2.94	2.05	0. 021	0.04	Small
Emotional symptoms of anxiety	20.03	5.87	21.69	5.34	1.73	0.043	0.03	Small
Total score	54.71	14.03	58.20	13.24	1.48	0.070	0.03	Small

It is clear from the values recorded in Table 10, it turns out that there are no statistically significant differences between the averages of the study sample scores on the COVID-19 vaccination anxiety scale, and the factor of (anxiety related to confidence in the effectiveness of the vaccine) according to the affliction of chronic diseases or not, where two values (*t*) were not statistically significant, while there were statistically significant differences between the two study groups in the factors of (anxiety related to professional, social and family responsibility, and emotional symptoms of anxiety), where two values (*t*) were significant at (0.05).

**The fourth assumption**: "the average scores of the study sample from faculty members at ASU on the scale of vaccination anxiety with COVID-19 vaccine and its sub-factors do not differ depending on the academic major (literary—academic)". To verify this assumption, a *t*-test was used for independent samples, and the results are listed in Table 11.

**Table 11:** Means, standard deviations, *t*-test values, and significance between faculty members in literary and academic major on the scale of COVID-19 vaccine anxiety and its sub-factors.

Statistical values	Literary specializations (*n* = 92)		Scientific specializations (*n* = 47)		t Value	Significance
Variable	Mean	Standard deviation	Mean	Standard deviation		
Anxiety related with vaccine confidence.	24.93	6.55	24.53	6.33	0.349	0.364
Anxiety related to professional, social, and family responsibilities.	10.87	2.99	11.11	2.97	0.442	0.329
Emotional symptoms of anxiety.	20.69	5.43	21.59	5.59	0.895	0.186
Total score	56.49	13.33	57.23	14.34	0.299	0.383

It is clear from the values recorded in Table 11, it turns out that there are no statistically significant differences between the averages of the study sample scores on the COVID-19 vaccination anxiety scale and its sub-factors according to the academic specialization; where all the values of (*t*) were non-statistically significant.

**The fifth assumption**: "The average scores of the study sample from faculty members at ASU on the COVID-19 vaccine vaccination anxiety scale and its sub-factors do not differ from the previous COVID-19 infection or not". To verify this assumption, a *t*-test was used for independent samples, and the results are listed in Table 12.

**Table 12:** Means, standard deviations, *t*-test values, and significance between faculty members with and without previous COVID-19 infection on the scale of COVID-19 vaccine anxiety and its sub-factors.

Statistical values	Previous COVID-19 infection (*n* = 59)		Non previous COVID-19 infection (*n* = 80)		t Value	Significance	D	Effect size
Variable	Mean	Standard deviation	Mean	Standard deviation				
Anxiety related with vaccine confidence	26.32	5.87	23.67	6.61	2.44	0.008	0.04	Small
Anxiety related to professional, social, and family responsibilities	11.37	2.92	10.64	2.99	1.44	0.075	0.02	Small
Emotional Symptoms of Anxiety	21.83	5.51	20.39	5.63	1.51	0.067	0.03	Small
Total score	59.52	12.8	54.70	13.94	2.088	0.019	0.04	Small

It is clear from the values recorded in Table 12, there are statistically significant differences between the averages of the study sample scores on the COVID-19 vaccination anxiety scale and the factor of (anxiety related to confidence in the effectiveness of the vaccine) according to the presence of a previous COVID-19 infection or not. Two values (t) were statistically significant at the significance level (0.01), with the differences being in the direction of those who had previously been infected with the epidemic, while it turned out that there were no statistically significant differences between the two study groups in the factors of (anxiety related to professional, social and family responsibility, and emotional symptoms of anxiety), where the values of (*t*) were not statistically significant.

**The sixth assumption**: "The average scores of the study sample from faculty members at ASU on the COVID-19 vaccine vaccination anxiety scale and its sub-factors do not differ according to gender (males- females)". To verify this assumption, a Mann–Whitney test was used for independent samples, and the results are listed in Table 13.

**Table 13:** Mean, mean of ranks, sum of ranks, z values, and significance between faculty members on the scale of COVID-19 vaccine anxiety and its sub-factors according to gender (males-females).

Values	Males (*n* = 20)			Females (*n* = 119)			Z Value	Sig.
Variable	Mean	Mean of ranks	Sum of ranks	Mean	Mean of ranks	Sum of ranks		
Anxiety related with vaccine confidence.	22.30	54.30	1086	25.22	72.64	8644	1.887	0.059
Anxiety related to professional, social, and family respons-ibilities	9.70	53	1060	11.15	72.86	8670	2.052	0.040
Emotional symptoms of anxiety	19.75	61.25	1225	21.21	71.47	8505	1.052	0.293
Total score	51.75	54.50	1110	57.58	72.44	8620	1.741	0.082

As shown in Table 13 a review of the mean ranks of the study sample on the COVID-19 vaccination anxiety scale and its dimensions, indicates that there are no statistical significant differences between the two study groups (Males- Females) on the COVID-19 vaccination anxiety scale and its sub-factors according to gender, except the factor of anxiety related to professional, social, and family responsibilities, (*z*) value (2.05) was statistically significant at 0.05 towards females.

## Discussion

As shown in Table 8, by reviewing the values of the partial correlation coefficient – which were squared – which represent the distinct contribution of each variable after deleting or excluding any common overlap or contrast with other variables, it becomes clear that the conspiracy beliefs variable makes a distinct contribution to (52%) of the explanation of the variation in the variable of vaccination anxiety with COVID-19 vaccine, followed by suggestibility (20%), then psychological flexibility (14%), and finally psychasthenia (12%)^13^.

This has been stated as follows: about the suggestibility variable and the significance of its contribution to predicting the vaccination anxiety variable; this result is consistent with the results of Killgore et al.^14^ study which revealed that both heightened fears of contracting COVID-19 and reduced psychological flexibility significantly predicted a greater desire to get vaccinated, and that 46.2% (of the USA study sample (*n* = 1062, aged 18–91 years with an average of 37 years) expressed fears about vaccination with the vaccine; political ideology was by far the most consistent predictor of both the desire to vaccinate and the fear of it, the results also indicated that to support the large-scale vaccination campaign and its success, frontline healthcare workers should tailor their discussions about the benefits of COVID-19 vaccination to align with each patient’s unique political and social perspectives^14^.

This result can be explained in light of the importance of the suggestibility variable and its impact on various aspects of an individual’s life, starting from his use of various media, social networking sites, passing through the rumors he receives from the media, from his colleagues at work or the general community, and watching it on TV or advertising, or what they hear from the news without trying to verify the sincerity of their beliefs. Many people tend to believe what is announced by a person of status or authority, and this effect is not limited to the psychological aspect only, but pushes us to simulate his actions, behavior, expressive movements, gestures, and voices^15^.

All these examples and many others were manifested during the corona virus pandemic as well as vaccination and its effects, and the rumors about its harmful effect on the following generations, on the health of fetuses, and the possibility of complications. This was supported by the insistence of some employers that vaccination should be compulsory without leaving the freedom of choice to the individual^16^. In Egyptian society, we find that some saw it as a conspiracy to achieve political and economic goals, as a cause of infertility, or as a way to get rid of the elderly, which was a cause for concern about vaccination in various social contexts.

In just one year, the virus that causes COVID-19 appeared on the scene, infecting almost 99 million people around the world, with more than 400,000 deaths recorded in the United States alone. Remarkably, many vaccines were developed quickly, rigorously tested, and fast-tracked for approval in less than a year, with the first doses administered in the United States on December 14, 2020. The primary goal of the immunization process was to vaccinate enough of the population to reach a state of “herd immunity” to reduce virus transmission, and to achieve this goal was for the public to be well informed and to collectively support vaccination efforts.

Several factors, including the unusually rapid pace of vaccine development, rapid approval, and the politicization of pandemic response efforts, have raised public doubts about the safety and potential effectiveness of newly developed vaccines. Thus, understanding the reasons for vaccine hesitancy and ways to foster trust among the public is vital to the success of vaccination efforts^14^.

As for the ability of the conspiracy belief variable to predict vaccination anxiety, this is consistent with the results of Debski et al.^17^ study, which examined conspiratorial theories during the coronavirus pandemic, and found a positive and significant correlation between the tendency to believe in general conspiracy theories and severity of anxiety about the COVID-19 pandemic among the study sample, as well as the study of Arshad et al.^18^ and his colleagues, who conducted a national survey to assess COVID-19 conspiracy beliefs among the general public in Pakistan and found the prevalence of vaccine-related conspiracy beliefs ranging from 9.3% to 28.4% among the study participants. The presence of these beliefs is associated with a decrease in vaccine acceptance, which represents a serious threat to the successful vaccination process^18^. It also agrees with the results of Al-Sanafi and Sallam’s^19^ study, which resulted in vaccine hesitancy being significantly associated with embracing vaccine conspiracy beliefs.

This can be explained in light of the difficulty of obtaining confirmed information about the epidemic, its causes, and consequently, the possibility of its cure, as well as the speed of its spread, conflicting information and opinions about it even among specialists, as well as the abundance of media channels, in addition to social media and conflicting facts and information.

This, in addition, stems from the individual’s desire to know and feel safe and confident in oneself and in the group to which he belongs, regarding the abundance of unreliable news and disregard for neutrality and logic intentionally or unintentionally. Moreover, we see the apparent contradiction in the results of academic research considering the causes of the corona virus pandemic. All this created a kind of need for “knowledge” to control anxiety, which led to the trend towards conspiratorial thinking and fabricating reasons and analyses to satisfy the need of individuals for knowledge and the completeness of their picture^20^.

Conspiracy beliefs have been associated with this pandemic early since these beliefs revolved around aspects related to the virus being man-made. Moreover, these harmful beliefs have extended to include concepts about possible vaccines, such as accusations of conspiracies to force vaccination, which would be used to implant microchips to control humans. There are additional claims that COVID-19 vaccines can cause infertility with the aim of limiting population growth. Such claims, without any evidence, are circulating on some social media platforms that can have tremendous negative impacts on the attitude of the general public toward vaccines^7,16^.

COVID-19 pandemic has led to mass conspiracy theories about its origins, as well as a widespread concern about the level of adherence to preventive measures. The results of Alper et al.^21^ study reported that a higher belief in intuition, uncertainty avoidance, and conspiracy beliefs were associated with a higher level of belief in a conspiracy theory regarding COVID-19. Besides, there are two important factors to predict belief in conspiracy theories; these are feelings of lacking control and uncertainty and self-uncertainty, which make conspiracy theories, seem to be more plausible and increase the tendency to believe them. A related factor is also the lack of control, which is positively related to the presence of plot mentality^21^.

People with high levels of uncertainty avoidance are also less tolerant to the mystery surrounding the pandemic (for example: the exact source of the disease, discussions about how it happened, how it can be cured, etcetera.). Avoiding this uncertainty as well as the anxiety it produces would make people more likely to believe in conspiracy theories, that offer “clearer” explanations, but they are incorrect. Since the feeling of stress and stressful life events such as illness and bereavement predict belief in conspiracy theories, the risk of COVID-19 can be an important indicator as well. In other words, people with a higher perception would increase conspiracy beliefs and make them dually predict vaccination anxiety.

As for the variable of psychasthenia and its insignificant contribution to predicting vaccination anxiety with the COVID-19 vaccine in the study sample, this can be explained in the light of what Samal^22^ pointed out that the reluctance to take vaccines is a complex phenomenon, and its context varies with time, place and vaccines. The model of reluctance to take vaccines consists of three basic points: confidence, satisfaction, and comfort. It is clear that a high level of vaccine hesitancy leads to a decrease in demand for the vaccine; the outbreak of many preventable diseases is associated with a reluctance to take the vaccine. This crisis is mainly man-made despite the unequivocal fact that vaccination is still the most important public health intervention of our time^22^.

Given this context, it is misinformation that will severely impact the vaccination efforts against the virus. Many surveys conducted in the recent past show a wide range of reluctance to take the vaccine among different populations in the world. One of the recent surveys conducted by the US Public Health Authority found that 12% of the US population does not want to take the vaccine, 82% of them are worried about the safety of the vaccine, and in another study conducted on university students in Italy, found that a student out of ten students did not show a positive reaction to take the vaccine, and 13.9% of the students hesitated in taking the vaccination, in an online survey in the UK (*n* = 1088) and Turkey (*n* = 3936) revealed that 14% and 31% of study participants in the UK and Turkey – respectively – were not sure about accepting a possible vaccine for COVID-19, and 3% of study participants refused to immediately accept the vaccine.

As for the hypothesis that the psychological flexibility variable does not significantly contribute to predicting COVID-19 vaccination anxiety in the study sample, this result is consistent with the results of Killgore et al.^14^ study, that a decrease in psychological flexibility significantly predicts the desire to take the vaccine^14^, and agrees with the results of the Kheirabadi et al.^23^ study, which explained that vaccine acceptance or reluctance to take it is of great importance for public health and that understanding the main factors affecting vaccine acceptance can help in developing strategies to improve the vaccination schedule. Therefore, the examined correlation of psychological flexibility with acceptance and reluctance to take the vaccine in a sample of 461 Iranians found a positive correlation between psychological flexibility and vaccine acceptance and it explained 14% of the variation in COVID-19 vaccine acceptance^23^.

However, it contradicts the results of Kimhi et al.^24^ study, which examined the relationship between psychological flexibility and the feeling of danger due to COVID-19, and showed a negative correlation between individual/community flexibility, feeling of danger and symptoms of distress. Individual flexibility was more able to predict the feeling of danger and symptoms of distress^24^.

As shown in Table 9, a review of the *t*-values indicates that these results are consistent with the findings of Al Wahibiya et al.^25^, which showed that individuals over the age of 40 are less anxious than others, likely due to the maturity of individuals at this age^25^. However, these results are inconsistent with the findings of Killgore et al.^14^ and Jennings et al.^7^, which indicated that aging significantly predicts the desire to take the COVID-19 vaccine^7,14^.

This result can be explained in light of the fact that individuals over the age of fifty may be more mature and experienced with various life adversities, more preoccupied with health and life matters than they are worried about hunger and vaccination itself, and individuals under the age of fifty are still in the prime of life; especially that the majority of them are in the age group from 27 to 40 years, and they still have many desires and goals that they want to achieve and events and pleasures that they have not lived yet.

As shown in Table 10, a review of the *t*-values indicates that these differences were towards non-chronically ill people, and this result is consistent with the results of Bendau et al.^26^ study which indicated that COVID-19 related anxiety of infection and health-related outcomes have been positively and significantly associated with acceptance of vaccination. In contrast, socioeconomic fears have been negatively associated with acceptance of vaccination^26^; however, they differ from the results of Sallam et al.^16^ study which indicated that those with a history of chronic diseases have higher rates of acceptance of COVID-19 vaccine. There is no statistical significant differences between the two study groups in the overall score on the COVID-19 vaccination anxiety scale can be explained in the light of the high rates of anxiety in general about the vaccine, its effectiveness and safety, especially with its rapid development and insufficient testing and with conflicting opinions about its safety, especially in the long term and the spread of a lot of rumors about it^16^.

As shown in Table 11, a review of the t-values indicates that these results are consistent with the results of Al-Otaibi and Abdul Raziq’s^27^ study, which showed that there are no differences between people with scientific and literary academic specialization in anxiety about corona virus; they explained this in the light of the fact that the virus represents a new epidemic that did not exist before^27^. In the current study, this can be explained in light of the lack of information about the virus, its development, and how to control it even among specialists, as well as the speed of vaccine production without much testing, which supports its safety and accurately explains its side effects in the near and long term, which has caused concern among all individuals of different academic specialties.

As shown in Table 12, a review of the t-values indicates that these results are differs with those of Killgore et al.^14^ study that the pre-diagnosis of COVID-19 significantly predicts the desire to get vaccinated^14^, the high rate of vaccination anxiety (as a total degree as well as the factor of confidence in the effectiveness of the vaccine) in those who have previously been infected with the epidemic can be explained by their suffering from the epidemic and its severe symptoms, pain and concern for their lives as a result of previous infection; as well as differing opinions about the effectiveness of the vaccine, their intense fear of the disease returning and the return of symptoms and suffering, and the likelihood of worsening its side effects compared to those who have not previously been infected.

As shown in Table 13, a review of the (z) values indicates that females are more anxious than males on the factor of anxiety related to professional, social, and family responsibilities; this can be explained by considering the nature of women in Eastern societies in general, and Egyptian society in particular. Females are often preoccupied with their families and have numerous family and social responsibilities. They are constantly attentive to every detail concerning their family members, taking on all responsibilities, from caring for children, supervising their upbringing, and monitoring their education and well-being. In addition to their duties as faculty members at a university, where they bear the responsibilities of teaching, scientific research, and administrative tasks related to their work.

Generally, the relevance of our work to understand the psychological factors that influence vaccination anxiety can help develop strategies aimed at reducing this anxiety and increasing vaccine acceptance, thus contributing to improved public health outcomes. Additionally, identifying the psychological variables associated with vaccination anxiety can facilitate the design of awareness campaigns that target the psychological issues affecting vaccine acceptance. The findings can shed light on communication methods and messaging related to vaccines.

## Conclusion

Variables of suggestibility and conspiracy beliefs have been found as the first and foremost predictors of COVID-19 vaccination anxiety. The aged over 50 years exhibited less rate of vaccination anxiety, more desire to take the COVID-19 vaccine, and moreover, a high rate of vaccination anxiety was observed in faculty members who have previously been infected with the epidemic and it does not differ according to the presence or absence of chronic diseases and academic specialization. Though both predictors are complex and may be influenced by many social and cultural factors, given the potential return of COVID-19 threat and other future health pandemic threats to our world, we must rethink and develop ways to reinforce them. Some future studies can be proposed as follows: 1) Developing positive thinking to reduce conspiracy beliefs associated with the spread of epidemics among various sectors of Egyptian society. 2) A comparative study of those who received vaccination and those who refused it related to the big five factors of personality.

### Limitations and implications

The results of the present study must be viewed within the context of specific limitations. The use of self-report scales, the sample size with an imbalance in the male-to-female ratio, and the sample being limited to faculty members at Ain Shams University may all affect the generalizability of the findings. The scales for vaccination anxiety, psychasthenia, psychological flexibility, suggestibility, and conspiracy beliefs were prepared by the researchers and have not been documented in previous studies. This highlights the need to reuse the prepared scales in future survey studies for documentation. Despite these limitations, the results are relevant and add to the evidence base in characterizing the role of some psychological and demographic variables in predicting vaccination anxiety even though the results showed a correlation between some of these variables and COVID-19 vaccination anxiety. Future studies among larger samples should replicate and expand the study.

## Data Availability

The data that support the findings of this study are available from the corresponding author upon reasonable request.
